# ﻿Note on the genus *Nemania* (Xylariaceae) – first records and a new species of the genus from Iran

**DOI:** 10.3897/mycokeys.93.94148

**Published:** 2022-10-07

**Authors:** Mohammad Javad Pourmoghaddam, Christopher Lambert, Hermann Voglmayr, Seyed Akbar Khodaparast, Irmgard Krisai-Greilhuber, Marc Stadler

**Affiliations:** 1 Department of Plant Protection, Faculty of Agricultural Sciences, University of Guilan, Rasht, Iran University of Guilan Rasht Iran; 2 Department Microbial Drugs, Helmholtz-Centre for Infection Research GmbH, Inhoffenstraße 7, 38124 Braunschweig, Germany Department Microbial Drugs, Helmholtz-Centre for Infection Research GmbH Braunschweig Germany; 3 Department for Molecular Cell Biology, Helmholtz-Centre for Infection Research GmbH, Inhoffenstraße 7, 38124 Braunschweig, Germany Department for Molecular Cell Biology, Helmholtz-Centre for Infection Research GmbH Braunschweig Germany; 4 Department of Botany and Biodiversity Research, University of Vienna, Rennweg 14, 1030 Wien, Austria University of Vienna Wien Austria; 5 Institute of Microbiology, Technische Universität Braunschweig, Spielmannstraße 7, 38106 Braunschweig, Germany Technische Universität Braunschweig Braunschweig Germany

**Keywords:** Ascomycota, molecular phylogenetics, *Nemaniaserpens*, one new species, Sordariomycetes, taxonomy, Xylariales

## Abstract

In a survey of xylarialean fungi in northern Iran, some specimens attributable to the genus *Nemania* were collected, cultured and sequenced. Morphological evidence and phylogenetic analyses of a combined ITS, LSU, *RPB2* and *TUB2* gene dataset confirmed the presence of *Nemaniadiffusa* and *N.serpens* in Iran for the first time. Furthermore, the new species *N.hyrcana*, which shows similarities to *N.subaenea* and its putative synonym *N.plumbea*, but significantly differs from the latter in its DNA sequences, was encountered. All species are illustrated, described and discussed. In the phylogenetic analyses, for the first time, the overlooked ex-type ITS sequences of the neotype of the generic type, *N.serpens* and that of the holotype of *N.prava*, were added to a multi-gene matrix of *Nemania*. This revealed that the two accessions of *N.serpens* (HAST 235 and CBS 679.86), for which multigene data are available in GenBank, are misidentified, while the Iranian accession of *N.serpens* has an almost identical ITS sequence to the neotype, confirming its morphological species identification. The two previously accepted species of *Euepixylon*, *E.udum* and *E.sphaeriostomum*, are embedded within *Nemania* and are revealed as close relatives of *N.serpens*, supporting the inclusion of *Euepixylon* in *Nemania*.

## ﻿Introduction

The genus *Nemania* S. F. Gray was established by Gray (1821) and has always been considered to belong to the family Xylariaceae Tul. & C. Tul., even though its species were placed in *Hypoxylon* for some time, according to the generic concepts established by Miller (1961) and other authors. The reason for this was that *Nemania* species superficially resemble those of *Hypoxylon* in having effused-pulvinate stromata on dead wood. Gray (1821) had used a somewhat ill-defined concept for this genus, which was resolved by Donk (1964) who selected *Sphaeriaserpens* as the type of the genus. Later, Pouzar (1985a, 1985b) emended *Nemania* and separated the genus from *Hypoxylon* according to morphological characters and Petrini and Rogers (1986) confirmed this by studies on the cultures, pointing out the geniculosporium-like anamorph of *Nemania* species (vs. the nodulisporium-like anamorphs that are typical for *Hypoxylon* s. str.). The anamorph genus *Geniculosporium* had even eventually been erected, based on the conidial state of “*Hypoxylon*” (i.e. *Nemania*) *serpens* by Chesters and Greenhalgh (1964). This holomorphic concept has meanwhile been supported by molecular phylogenetic studies (e.g. Hsieh et al. 2010) that clearly revealed close affinities of *Nemania* to *Xylaria* and other genera with geniculosporium-like anamorphs. The most important monographs on the genus by Granmo et al. (1999) and Ju and Rogers (2002), however, still relied on morphological characters and many of the 37 taxa that were recognised by these authors have not yet been characterised by DNA sequence data.

*Nemania* is characterised by carbonaceous, superficial, multiperitheciate, effused-pulvinate stromata with papillate ostioles and variable presence of soft, whitish, brownish, grey or yellow internal tissue. Stromata do not release pigments in 10% potassium hydroxide (KOH). Asci are cylindrical, short or long stipitate, persistent, with an apical apparatus of various shapes, amyloid (like *N.diffusa*) or inamyloid (like *N.serpens*) in Melzer’s iodine reagent. Ascospores are pale brown to dark brown or blackish-brown, ellipsoidal, cylindrical or fusoid, inequilateral, slightly inequilateral or nearly equilateral, with acute, narrowly rounded or broadly rounded ends, with a straight, conspicuous or inconspicuous germ slit of spore length to much less than spore-length. It has geniculosporium-like anamorphs (Ju and Rogers 2002; Fournier et al. 2018).

During our survey of Xylariales specimens in northern Iran, three *Nemania* taxa were recorded. Species were identified, based on morphological and molecular phylogenetic analyses. As a result, a new species and records of two further species are reported from Iran, for which detailed morphological descriptions, illustrations and phylogenetic information are here provided.

## ﻿Materials and methods

### ﻿Morphological observation

The fungal specimens were collected in northern Iran (Guilan, Mazandaran and Golestan Provinces). For light microscopy, fresh collections, single ascospore isolations and cultures were examined for macro- and micromorphological characteristics, according to Ju and Rogers (2002) and Pourmoghaddam et al. (2018). Dried specimens were deposited in the University of Guilan Mycological Herbarium (**GUM**). Living cultures were deposited in the culture collection MUCL (Louvain la-Neuve, Belgium) and in the Iranian Fungal Culture Collection, Iranian Research Institute of Plant Protection, Tehran, Iran (**IRAN**).

### ﻿DNA extraction, PCR and sequencing

DNA extraction of fresh cultures and amplification of the ITS (nuc rDNA internal transcribed spacer region containing ITS1-5.8S-ITS2), LSU (5' 1200 bp of the large subunit nuc 28S rDNA), *RPB2* (partial second largest subunit of the DNA-directed RNA polymerase II) and *TUB2* (partial β-tubulin) loci were carried out as described by Wendt et al. (2018).

### ﻿Phylogenetic analyses

Published sequences of a single accession for each *Nemania* species served as basis for the sequence matrix. Information on all used strains, their corresponding sequences and GenBank accession numbers can be found in Table 1. In addition to the sequences retrieved from GenBank, ITS sequences of the holotype of *N.prava* and of the neotype of *N.serpens* were manually transcribed from the ITS alignment published as colour figure appendix 3 in Granmo et al. (1999), because these sequences have not been deposited in a public sequence repository. In addition, to have the ITS sequences of Granmo et al. (1999) available for further studies, the transcribed ex-type sequences were also submitted to GenBank (ex-neotype sequence of *N.colliculosa*: OP289676, ex-holotype sequence of *N.prava*: OP289674, ex-neotype sequence of *N.serpens*: OP289675). To reveal the phylogenetic position of the Iranian *Nemania* accessions, the newly-generated sequences were aligned with the GenBank sequences. All alignments were produced with the server versions of MAFFT v. 7.490 (www.ebi.ac.uk/Tools/mafft or http://mafft.cbrc.jp/alignment/server/; Katoh et al. 2019) and checked and refined using BioEdit v. 7.0.4.1 (Hall 1999).

**Table 1.** Isolation and accession numbers of sequences used in the phylogenetic analyses. Isolates/sequences in bold were isolated/sequenced in present study. N/A: not available.

Species	Strain number	Origin	Status	GenBank accession numbers				Reference
				ITS	LSU	*RPB2*	*TUB2*	
*Amphiroselliniafushanensis*	HAST 91111209	Taiwan	HT	GU339496	N/A	GQ848339	GQ495950	Hsieh et al. (2010)
*Amphirosellinianigrospora*	HAST 91092308	Taiwan	HT	GU322457	N/A	GQ848340	GQ495951	Hsieh et al. (2010)
*Anthostomelloideskrabiensis*	MFLUCC 15-0678	Thailand	HT	KX305927	KX305928	KX305929	N/A	Tibpromma et al. (2017)
*Astrocystisconcavispora*	MFLUCC 14.0174	Italy		KP297404	KP340545	KP340532	KP406615	Daranagama et al. (2015)
*Biscogniauxianummularia*	MUCL 51395	France	ET	KY610382	KY610427	KY624236	KX271241	Wendt et al. (2018)
*Clypeosphaeriamamillana*	CBS 140735	France	ET	KT949897	KT949897	MF489001	N/A	Jaklitsch et al. (2016), Voglmayr et al. (2018)
*Collodisculabambusae*	GZU H0102	China		KP054279	KP054280	KP276675	KP276674	Li et al. (2015)
*Collodisculafangjingshanensis*	GZU H0109	China	HT	KR002590	KR002591	KR002592	KR002589	Li et al. (2015)
*Collodisculajaponica*	CBS 124266	China		JF440974	JF440974	KY624273	KY624316	Jaklitsch and Voglmayr (2012), Wendt et al. (2018)
*Coniolarelialimoniispora*	MUCL 29409	Japan		MN984615	MN984624	MN987235	MN987240	Wittstein et al. (2020)
*Dematophorabunodes*	CBS 123597	Peru		MN984619	MN984625	N/A	MN987245	Wittstein et al. (2020)
*Dematophorabuxi*	JDR 99	France		GU300070	N/A	GQ844780	GQ470228	Hsieh et al. (2010)
*Dematophoranecatrix*	CBS 349.36	Argentina		AY909001	KF719204	KY624275	KY624310	Peláez et al. (2008), Wendt et al. (2018)
*Dematophorapepo*	CBS 123592	Peru		MN984620	N/A	N/A	MN987246	Wittstein et al. (2020)
*Entoleucamammata*	JDR 100	France		GU300072	N/A	GQ844782	GQ470230	Hsieh et al. (2010)
*Graphostromaplatystomum*	CBS 270.87	France	HT	JX658535	DQ836906	KY624296	HG934108	Stadler et al. (2014), Zhang et al. (2006), Wendt et al. (2018), Koukol et al. (2015)
*Hypocreodendronsanguineum*	JDR 169	Mexico		GU322433	N/A	GQ844819	GQ487710	Hsieh et al. (2010)
*Hypoxylonfragiforme*	MUCL 51264	Germany	ET	KC477229	KM186295	KM186296	KX271282	Stadler et al. (2013), Daranagama et al. (2015), Wendt et al. (2018)
*Hypoxylonhoweanum*	MUCL 47599	Germany		AM749928	KY610448	KY624258	KC977277	Bitzer et al. (2008), Kuhnert et al. (2014), Wendt et al. (2018)
*Kretzschmariaclavus*	YMJ 114	French Guiana		EF026126	N/A	GQ844789	EF025611	Hsieh et al. (2010)
*Kretzschmariadeusta*	CBS 163.93	Germany		KC477237	KY610458	KY624227	KX271251	Stadler et al. (2013), Wendt et al. (2018)
*Kretzschmariadeusta*	CBS 826.72	Belgium		KU683767	KU683767	KU684309	KU684190	U’Ren et al. (2016)
***Kretzschmariadeusta***	**MUCL 57705**	**Iran**		MH084755	**OP359327**	**OP359596**	**OP359601**	Pourmoghaddam et al. (2018), **This study**
***Kretzschmariahedjaroudei***	**MUCL 57706**	**Iran**	**HT**	MH084757	**OP359328**	**OP359597**	**OP359602**	Pourmoghaddam et al. (2018), **This study**
*Kretzschmariaguyanensis*	HAST 89062903	Taiwan		GU300079	N/A	GQ844792	GQ478214	Hsieh et al. (2010)
*Kretzschmarialucidula*	YMJ 112	French Guiana		EF026125	N/A	GQ844790	EF025610	Hsieh et al. (2010)
*Kretzschmariamegalospora*	YMJ 229	Malaysia		EF026124	N/A	GQ844791	EF025609	Hsieh et al. (2010)
*Kretzschmarianeocaledonica*	HAST 94031003	Taiwan		GU300078	N/A	GQ844788	GQ478213	Hsieh et al. (2010)
*Kretzschmariapavimentosa*	JDR 109	Taiwan		GU300077	N/A	GQ844787	GQ478212	Hsieh et al. (2010)
*Kretzschmariasandvicensis*	JDR 113	USA		GU300076	N/A	GQ844786	GQ478211	Hsieh et al. (2010)
*Linosporopsisischnotheca*	CBS 145761	Switzerland	ET	MN818952	MN818952	MN820708	MN820715	Voglmayr and Beenken (2020)
*Linosporopsisochracea*	CBS 145999	Germany	ET	MN818958	MN818958	MN820714	MN820721	Voglmayr and Beenken (2020)
*Nemaniaabortiva*	BISH 467	USA	HT	GU292816	N/A	GQ844768	GQ470219	Hsieh et al. (2010)
*Nemaniaaquilariae*	KUMCC 20-0268	China	HT	MW729422	MW729420	MW717891	MW881142	Tibpromma et al. (2021)
*Nemaniabeaumontii*	HAST 405	Martinique		GU292819	N/A	GQ844772	GQ470222	Hsieh et al. (2010)
*Nemaniabipapillata*	HAST 90080610	Taiwan		GU292818	N/A	GQ844771	GQ470221	Hsieh et al. (2010)
*Nemaniacamelliae*	GMB0068	China	HT	MW851889	MW851872	MW836055	MW836029	Pi et al. (2021)
*Nemaniacaries*	GMB0070	China		MW851874	MW851857	MW836071	MW836036	Pi et al. (2021)
*Nemaniachangningensis*	GMB0056	China	HT	MW851875	MW851858	MW836061	MW836027	Pi et al. (2021)
*Nemaniachestersii*	JF 04024	France		N/A	DQ840072	DQ631949	DQ840089	Tang et al. (2007; 2009)
*Nemaniacyclobalanopsina*	GMB0062	China	HT	MW851883	MW851866	MW836057	MW836025	Pi et al. (2021)
*Nemaniadelonicis*	MFLU 19-2124	Thailand	HT	MW240613	MW240542	MW342617	MW775574	Samarakoon et al. (2022)
*Nemaniadiffusa*	HAST 91020401	Taiwan		GU292817	N/A	GQ844769	GQ470220	Hsieh et al. (2010)
*Nemaniaethancrensonii*	CBS 148337	USA	HT	ON869311	ON869311	ON808489	ON808533	Voglmayr et al. (2022)
*Nemaniafeicuiensis*	GMB0059	China	HT	MW851880	MW851863	MW836063	MW836023	Pi et al. (2021)
*Nemaniafusoidispora*	GZUH0098	China		MW851881	MW851864	MW836070	MW836037	Ariyawansa et al. (2015)
***Nemaniahyrcana***	**MUCL 57704**	**Iran**	**HT**	**OP359332**	**OP359329**	**OP359598**	**OP359603**	**This study**
***Nemaniahyrcana***	**MUCL 57703**	**Iran**		**OP359333**	**OP359330**	**OP359599**	**OP359604**	**This study**
*Nemaniaillita*	YMJ 236	USA		EF026122	N/A	GQ844770	EF025608	Hsieh et al. (2010)
*Nemanialishuicola*	GMB0065	China	HT	MW851886	MW851869	MW836065	MW836033	Pi et al. (2021)
*Nemanialongipedicellata*	MFLU 18-0819	Thailand	HT	MW240612	MW240541	MW342616	MW775573	Samarakoon et al. (2022)
*Nemaniamacrocarpa*	WSP 265	USA	HT	GU292823	N/A	GQ844776	GQ470226	Hsieh et al. (2010)
*Nemaniamaritima*	HAST 89120401	Taiwan	ET	GU292822	N/A	GQ844775	GQ470225	Hsieh et al. (2010)
*Nemaniaparaphysata*	MFLU 19-2121	Thailand	HT	MW240609	MW240538	MW342613	N/A	Samarakoon et al. (2022)
*Nemaniaplumbea*	JF TH-04-01	Thailand	HT	DQ641634	DQ840071	DQ631952	DQ840084	Tang et al. (2007; 2009)
*Nemaniaprava*	CBS 679.86	Switzerland	PT ^2^	KU683765	KU683765	KU684284	KU684188	U’Ren et al. (2016)
*Nemaniaprava*	TROM 104	Norway	HT	OP2896743	N/A	N/A	N/A	Granmo et al. (1999)
*Nemaniaprimolutea*	HAST 91102001	Taiwan	HT	EF026121	N/A	GQ844767	EF025607	Hsieh et al. (2010)
*Nemaniarubi*	GMB0064	China	HT	MW851885	MW851868	MW836059	MW836021	Pi et al. (2021)
*Nemaniaserpens*	TROM 174	Norway	NT	OP2896753	N/A	N/A	N/A	Granmo et al. (1999)
***Nemaniaserpens***	**MUCL 57702**	**Iran**		**OP359334**	**OP359331**	**OP359600**	**OP359605**	**This study**
*Nemaniaserpens*	HAST 235	Canada		GU292820	N/A	GQ844773	GQ470223	Hsieh et al. (2010)
*Nemaniasphaeriostoma*	JDR 261	USA		GU292821	N/A	GQ844774	GQ470224	Hsieh et al. (2010)
*Nemaniathailandensis*	MFLU 19-2117	Thailand	HT	MW240611	MW240540	MW342615	MW775572	Samarakoon et al. (2022)
*Nemaniauda*	CBS 148422	Austria	HT	ON869312	ON869312	ON808488	ON808532	Voglmayr et al. (2022)
*Nemaniayunnanensis*	KUMCC 20-0267	China	HT	MW729423	MW729421	MW717892	MW881141	Tibpromma et al. (2021)
*Podosordariamexicana*	WSP 176	Mexico		GU324762	N/A	GQ853039	GQ844840	Hsieh et al. (2010)
*Podosordariamuli*	WSP 167	Mexico	HT	GU324761	N/A	GQ853038	GQ844839	Hsieh et al. (2010)
*Poroniapileiformis*	WSP 88113001	Taiwan	ET	GU324760	N/A	GQ853037	GQ502720	Hsieh et al. (2010)
*Poroniapunctata*	CBS 656.78	Australia		KT281904	KY610496	KY624278	KX271281	Senanayake et al. (2015), Wendt et al. (2018)
*Roselliniaaquila*	MUCL 51703	France		KY610392	KY610460	KY624285	KX271253	Wendt et al. (2018)
Roselliniacf.akulovii	MUCL 57710	Iran		OL635184	OL635175	OL657210	OL657219	Pourmoghaddam et al. (2022)
Roselliniacf.akulovii	MUCL 57711	Iran		OL635185	OL635176	OL657211	OL657220	Pourmoghaddam et al. (2022)
*Roselliniacorticium*	MUCL 51693	France		KY610393	KY610461	KY624229	KX271254	Wendt et al. (2018)
*Roselliniacorticium*	STMA 13324	Germany		MN984621	MN984627	MN987237	MN987241	Wittstein et al. (2020)
*Roselliniacorticium*	MUCL 57714	Iran		OL635180	OL635171	OL657206	OL657215	Pourmoghaddam et al. (2022)
*Rosellinianectrioides*	CBS 449.89	Sweden		MN984622	MN984628	MN987239	N/A	Wittstein et al. (2020)
*Sarcoxyloncompunctum*	CBS 359.61	South Africa		KT281903	KY610462	KY624230	KX271255	Senanayake et al. (2015), Wendt et al. (2018)
*Stilbohypoxylonelaeicola*	Y.M.J 173	French Guiana		EF026148	N/A	GQ844826	EF025616	Hsieh et al. (2010)
*Stilbohypoxylonquisquiliarum*	Y.M.J 172	French Guiana		EF026119	N/A	GQ853020	EF025605	Hsieh et al. (2010)
*Xylariaacuminatilongissima*	HAST 95060506	Taiwan	HT	EU178738	N/A	GQ853028	GQ502711	Hsieh et al. (2010)
*Xylariaadscendens*	J.D.R 865	Thailand		GU322432	N/A	GQ844818	GQ487709	Hsieh et al. (2010)
*Xylariaarbuscula*	CBS 126415	Germany		KY610394	KY610463	KY624287	KX271257	Fournier et al. (2011), Wendt et al. (2018)
*Xylariabambusicola*	WSP 205	Taiwan	HT	EF026123	N/A	GQ844802	AY951762	Hsieh et al. (2010)
*Xylariabrunneovinosa*	HAST 720	Martinique	HT	EU179862	N/A	GQ853023	GQ502706	Hsieh et al. (2010)
*Xylariacurta*	HAST 494	Martinique		GU322444	N/A	GQ844831	GQ495937	Hsieh et al. (2010)
*Xylariadiscolor*	HAST 131023	USA	ET	JQ087405	N/A	JQ087411	JQ087414	Hsieh et al. (2010)
*Xylariahypoxylon*	CBS 122620	Sweden	ET	KY610407	KY610495	KY624231	KX271279	Sir et al. (2016), Wendt et al. (2018)
*Xylariamultiplex*	HAST 580	Martinique		GU300098	N/A	GQ844814	GQ487705	Hsieh et al. (2010)
*Xylariapolymorpha*	MUCL 49884	France		KY610408	KY610464	KY624288	KX271280	Wendt et al. (2018)

^1^ET ex-epitype, HT ex-holotype, NT ex-neotype, PT ex-paratype. ^2^ ex-paratype culture of Hypoxylonatropurpureumvar.brevistipitatum (= *Nemaniaprava*); deposited in GenBank as *Nemaniaserpens*. ^3^ ITS sequence originally not deposited in a sequence repository, but published as colour figure (Appendix 3) in Granmo et al. (1999); manually transcribed sequence deposited in GenBank in this study.

For the phylogenetic analyses, 90 accessions of 86 species of Xylariaceae and four outgroup taxa from Graphostromataceae (*Biscogniauxianummularia*, *Graphostromaplatystomum*) and Hypoxylaceae (*Hypoxylonfragiforme*, *H.howeanum*) were included. We also included the newly-sequenced LSU, *RPB2* and *TUB2* loci of the Iranian collections of *Kretzschmariahedjaroudei* (MUCL 57706) and *K.deusta* (MUCL 57705); for details on those accessions, see Pourmoghaddam et al. (2018). The sequence matrices of ITS, LSU, *RPB2* and *TUB2* were combined; after exclusion of ambiguously aligned and gappy regions, the resulting combined data matrix contained 4616 alignment positions from four loci (543 from ITS, 1275 from LSU, 1191 from *RPB2* and 1607 from *TUB2*).

Maximum Likelihood (ML) analyses were performed with RAxML (Stamatakis 2006) as implemented in raxmlGUI 1.3 (Silvestro and Michalak 2012) using the ML + rapid bootstrap setting and the GTRGAMMA substitution model with 1000 bootstrap replicates.

Maximum Parsimony (MP) analyses were performed with PAUP v. 4.0a169 (Swofford 2002). All molecular characters were unordered and given equal weight; analyses were performed with gaps treated as missing data; the COLLAPSE command was set to MINBRLEN. MP analysis of the combined multilocus matrix was done using 1000 replicates of heuristic search with random addition of sequences and subsequent TBR branch swapping (MULTREES option in effect, steepest descent option not in effect). Bootstrap analyses with 1000 replicates were performed in the same way, but using 10 rounds of random sequence addition and subsequent branch swapping during each bootstrap replicate. Bootstrap values ≤ 70% are considered low, between 70 and 90% intermediate and ≥ 90% high.

## ﻿Results

### ﻿Molecular phylogeny

**Figure 1.**
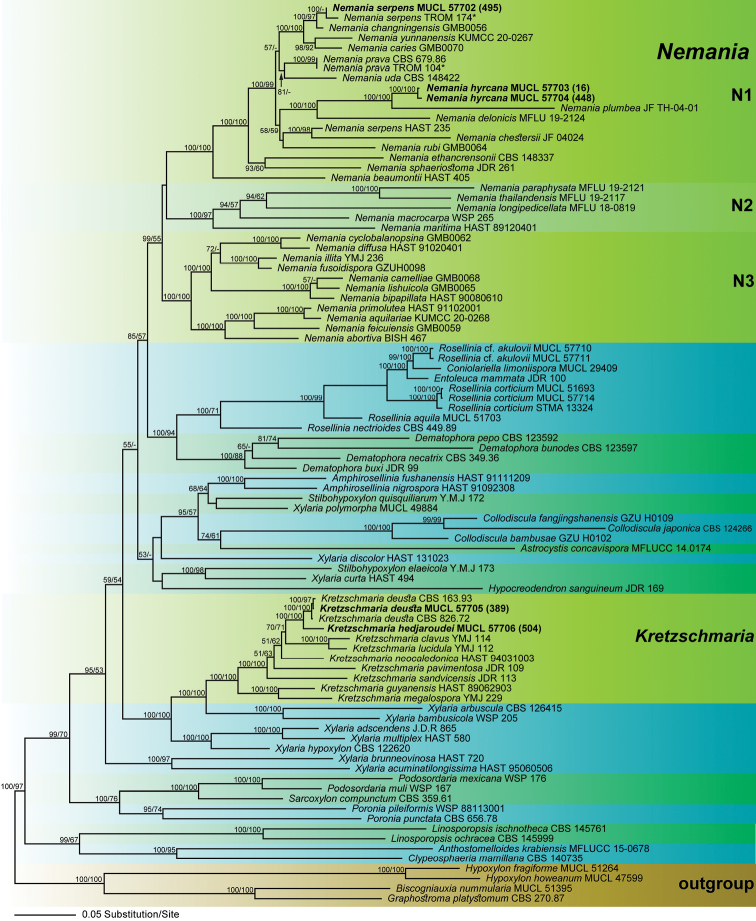
Phylogram of the best ML trees (lnL = −88,062.8606) revealed by RAxML from an analysis of the combined ITS-LSU-*RPB2*–*TUB2* matrix of selected Xylariaceae. Strains in bold were sequenced in the current study; for strains marked with an asterisk (*), ITS sequences were transcribed from Appendix 3 of Granmo et al. (1999). ML and MP bootstrap support above 50% are given at the first and second positions, respectively, above or below the branches.

Of the 4616 characters of the combined matrix, 1884 were parsimony informative (284 in ITS, 142 in LSU, 613 in *RPB2* and 845 in *TUB2*). The phylogram of the best ML tree (lnL = − 88,062.8606) obtained by RAxML is shown as Fig. 1. The MP analysis revealed two trees of length 20,490 (not shown) that had a similar topology to the ML tree. The phylogenies reveal a monophyletic clade of *Nemania* (including *Euepixylon*), like in previous studies (Wendt et al. 2018; Pi et al. 2021; Samarakoon et al. 2022; Voglmayr et al. 2022). Within Xylariaceae, the *Nemania* clade is most closely related to the genera *Coniolariella*, *Dematophora*, *Entoleuca* and *Rosellinia*.

The genus *Nemania* (including *Euepixylon*) receives high ML (99%), but low MP (55%) support and contains three highly-supported subclades (N1-N3 in Fig. 1). The ML and MP analyses reveal the same topologies within *Nemania*, except for minor differences (not shown). As these differences are not relevant within the context of our study, they are not further considered here.

The new *Nemania* species clustered together with *N.plumbea* (JF TH-04-01) with maximum ML and MP BS support, which is a sister group to *N.delonicis*, also with maximum ML and MP BS support (Fig. 1). The ITS sequence of the Iranian collection of *N.serpens* is almost identical to the ex-neotype sequence from *N.serpens* (TROM 174) from Granmo et al. (1999) and they clustered together with maximum ML BS support. The *N.serpens* clade has a sister group relationship with *N.changningensis* with maximum ML BS support. However, another isolate deposited as *N.serpens* (HAST 235) is not contained within the *N.serpens* clade, but remotely placed as sister species to *N.chestersii*, indicating a misidentification. The ex-holotype ITS sequence of *N.prava* (TROM 104) from Granmo et al. (1999) was almost identical to *N.serpens* (CBS 679.86) and both cluster together with maximum BS support; the latter was re-identified and given in the phylogenetic tree (Fig. 1) as *N.prava* (see discussion for details). Remarkably, the two previously accepted species of *Euepixylon*, the European *E.udum* and the North American *E.sphaeriostomum*, are placed within *Nemania* subclade N1, but are not revealed as closest relatives (Fig. 1), supporting their classification within *Nemania*.

### ﻿Taxonomy

#### ﻿ Nemania hyrcana

Taxon classificationFungiXylarialesXylariaceae

Pourmoghaddam, Voglmayr & Khodaparast sp. nov.

6BE3E0DD-9F56-585F-B7DB-45865F5536D9

845436

Figs 2 , 3

##### Holotype.

Iran, Guilan Province, Astara County, Darband Forest, 38°21'26"N, 48°50'19"E, 17 m elev., on fallen branch of *Parrotiapersica*, 7 October 2017, leg. M.J. Pourmoghaddam (GUM 1628; living culture MUCL 57704).

##### Etymology.

The epithet is derived from “Hyrcania”, an ancient biogeographical region, located in the south of the Caspian Sea where the specimens were collected.

##### Diagnosis.

differs from *Nemaniasubaenea* by its smaller ascospores [12–16 × 4.5–6 vs. 14–17.5 × 6–7.5 µm].

##### Teleomorph.

Stromata superficial, effused-pulvinate, up to 2.5 cm long, 0.2–1.4 cm wide, sessile, attachment to substrate with narrow connective; surface brown, dark brown, dark grey with a slightly shiny metallic tone, with conspicuous perithecial mounds; carbonaceous tissue immediately beneath the surface and between the perithecia; tissue beneath the perithecial layer conspicuous. Perithecia obovoid to spherical, 0.5–0.7 mm high × 0.4–0.6 mm wide; ostioles papillate to coarsely papillate. Asci cylindrical, with amyloid, urn-shaped apical apparatus, 3.5–4 µm high × 2.5–3 µm wide, stipe up to 130 µm long, spore-bearing part 60–85 × 8–12 µm. Ascospores smooth, unicellular, pale brown to brown, ellipsoid, inequilateral, with narrowly rounded ends, 12–16 × 4.5–6 µm, with straight germ slit much less than spore-length on dorsal side; perispore indehiscent in 10% KOH.

**Figure 2.**
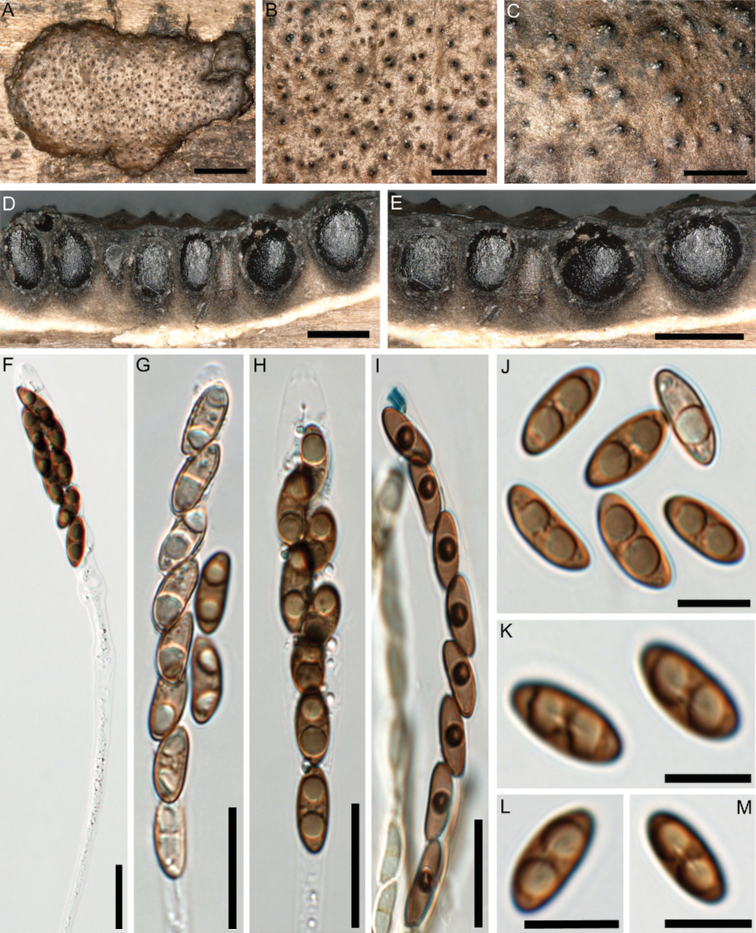
*Nemaniahyrcana* (Holotype GUM 1628) **A, B** close-up view of stromatal surface **C** close-up view of stromatal surface showing ostioles **D, E** stroma in horizontal section showing perithecia **F** mature ascus in water with long stipe **G** immature ascus in water **H** mature ascus in water **I** mature ascus in Melzer’s reagent **J** immature and mature ascospores in water **K–M** ascospores in water showing straight germ slit much less than spore-length. Scale bars: 2 mm (**A**); 0.8 mm (**B**); 0.5 mm (**C, E**); 0.4 mm (**D**); 20 µm (**F–I**); 10 µm (**J–M**).

##### Cultures and anamorph.

**Figure 3.**
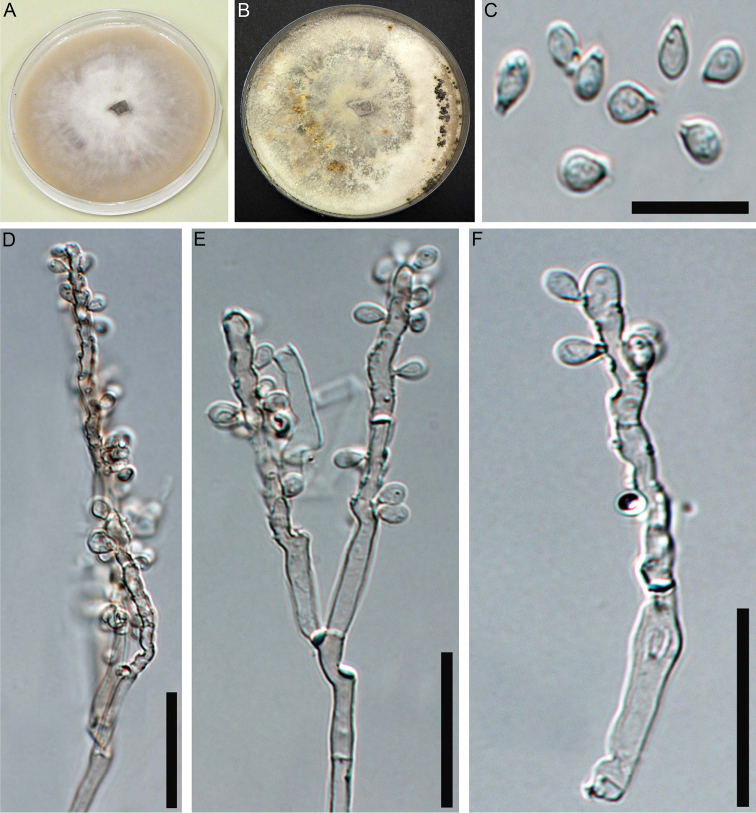
Culture and anamorphic structures of *Nemaniahyrcana* (MUCL 57704) on OA **A, B** surface of colony after (**A**) 7 and (**B**) 50 days of incubation **C** conidia **D–F** general view of anamorph structure, conidiophores, conidiogenous cells and mature conidia of *N.hyrcana*. Scale bars: 10 µm (**C**); 20 µm (**D–F**).

Colonies on OA covering a 9 cm Petri dish in 2 wk, at first white, becoming buff (45), felty, azonate; finally, attaining cream to grey after 50 days. Anamorph geniculosporium-like. Conidiophores variables in length, hyaline to light brown. Conidiogenous cells up to 50 × 2.5–3.5 µm, hyaline to light brown. Conidia hyaline, ellipsoid with truncate base, 3.5–6 × 2.5–3.5 µm (Fig. 3).

##### Other specimen examined.

Iran, Golestan Province, Aliabad-e-Katul County, Kaboudwall Forest, 36°52'25"N, 54°53'14"E, 1076 m elev., on dead branches (host unknown), 10 November 2017, leg. M.J. Pourmoghaddam (GUM 1627; living culture MUCL 57703, IRAN 3734C).

##### Notes.

**Figure 4.**
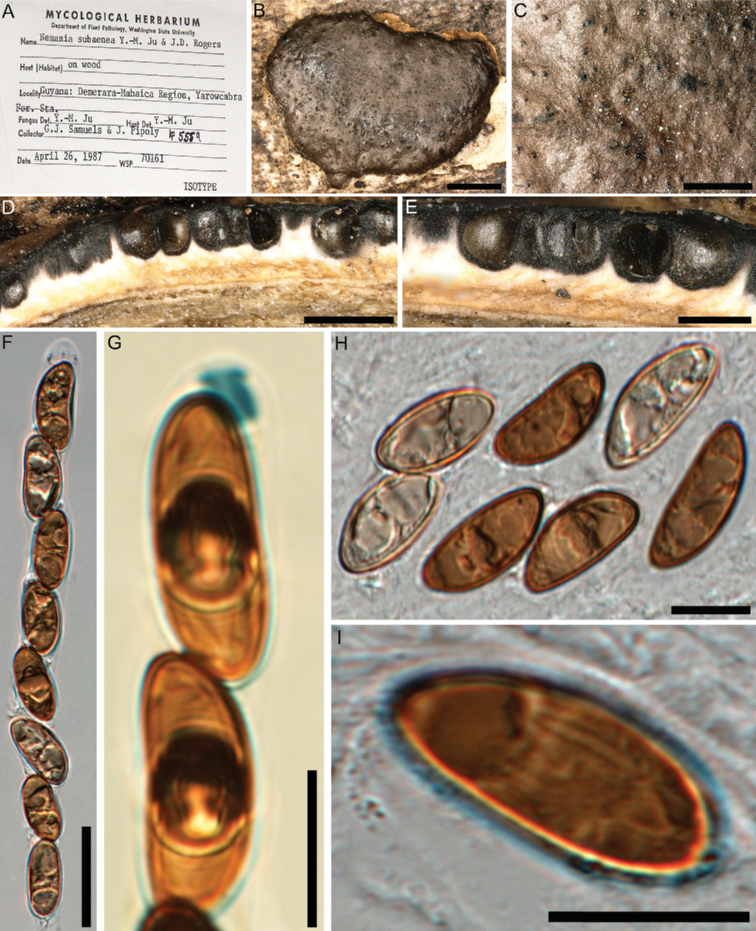
*Nemaniasubaenea* (isotype) **A** herbarium label **B** close-up view of stromatal surface **C** close-up view of stromatal surface showing ostioles **D, E** stroma in horizontal section showing perithecia **F** immature ascus in water **G** ascus apical plug in Melzer’s reagent **H** immature and mature ascospores in water **I** ascospore in water showing straight germ slit much less than spore-length. Scale bars: 2 mm (**B**); 0.8 mm (**C**); 1 mm (**D**); 0.5 mm (**E**); 20 µm (**F**); 10 µm (**G–I**).

This species resembles *Nemaniasubaenea* (Fig. 4), which was erected based on a single specimen from Guyana by Ju and Rogers (2002). Later, Fournier et al. (2018) reported it from Martinique and also mentioned *N.plumbea*, another single-specimen-based species from Thailand (Tang et al. 2007), which differs from *N.subaenea* only in the stromatal surface colour and in having slightly smaller ascospores (Tang et al. 2007). However, neither Ju and Rogers (2002) nor Fournier et al. (2018), who proposed that *N.plumbea* should be regarded as a synonym of *N.subaenea*, studied the cultures and anamorph of the neotropical species. The type of *N.plumbea*, on the other hand, was cultured and DNA sequences are available for comparison with the Iranian species. A comparison of these sequence data revealed significant differences between the two Iranian strains of *N.hyrcana* (MUCL 57703/ MUCL 57704) and the ex-type strain of *N.plumbea* (29/31 bp differences of 494 nucleotide characters in the ITS: 19/20 substitutions, 10/11 indels; 2 bp differences of 764 nucleotide characters in the LSU: 2 substitutions; 27 bp differences of 884 nucleotide characters in the *RPB2*: 28/27 substitutions; and 319/321 bp differences of 1422 nucleotide characters in the *TUB2*: 279/282 substitutions, 40/39 indels). This supports the erection of a new species for the Iranian fungus, for which multiple specimens and two cultures are available. Even if *N.plumbea* is not regarded as a synonym of *N.subaenea*, it should be kept in mind that both taxa are derived from tropical areas that are far away from Iran.

#### ﻿ Nemania serpens

Taxon classificationFungiXylarialesXylariaceae

(Pers.) Gray, Nat. Arr. Brit. Pl. (London) 1: 516 (1821).

A3BEBF11-E108-5C19-80A3-DD0A7DCAC3D6

Figs 5 , 6

##### Teleomorph.

Stromata superficial, effused-pulvinate, up to 4 cm long × 0.2–1.2 cm wide, sessile, attachment to substrate with strong connective; surface dark brown to black, with conspicuous perithecial mounds, carbonaceous immediately beneath surface; tissue between and beneath perithecia black to dark brown. Perithecia obovoid, 0.35–0.65 mm high × 0.25–0.4 mm wide, ostioles papillate to coarsely papillate. Asci cylindrical, stipe up to 130 µm long, spore-bearing part 55–70 × 7–9 µm, apical apparatus not bluing in Melzer’s reagent, dextrinoid (= red to red brown) in Lugol’s solution. Ascospores smooth, unicellular, pale brown to brown, ellipsoid, inequilateral, with narrowly or broadly rounded ends, 10–14 × 4–5(–6) µm, with straight germ slit much less than spore-length; perispore indehiscent in 10% KOH.

**Figure 5.**
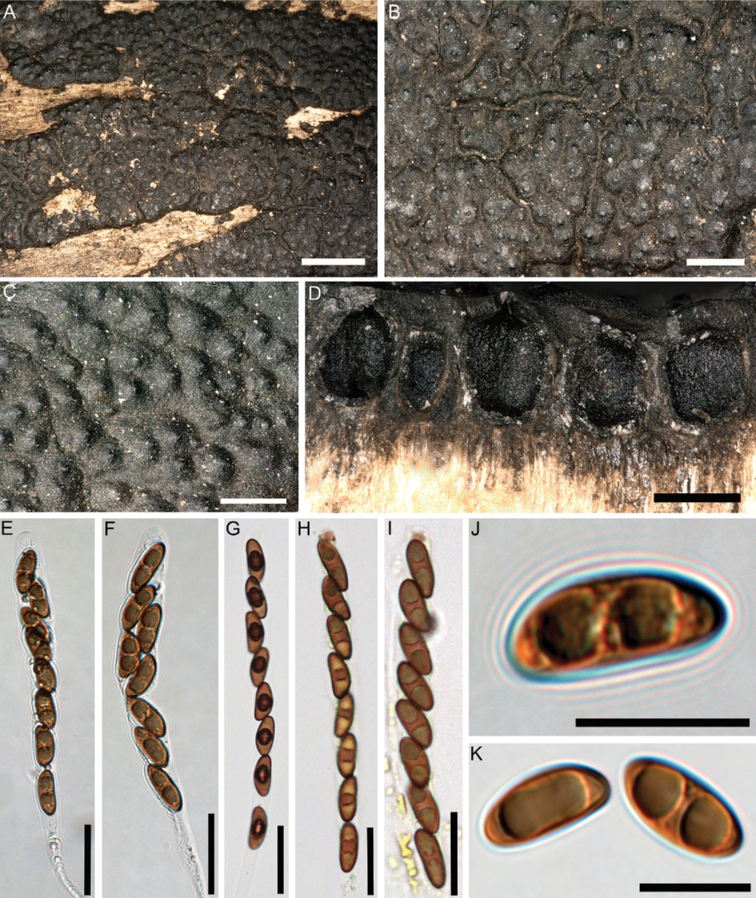
*Nemaniaserpens* (GUM 1625) **A, B** close-up view of stroma surface **C** close-up view of stroma surface showing ostioles **D** stroma in vertical section showing perithecia **E, F** mature asci in water **G** mature ascus in Melzer’s reagent, showing the inamyloid (not bluing) ascal apical apparatus **H, I** mature ascus in Lugol’s solution, showing the dextrinoid (= red to red brown) ascal apical apparatus **J** ascospore in water **K** ascospores in water showing straight germ slit much less than spore-length. Scale bars: 3 mm (**A**); 1 mm (**B**); 0.6 mm (**C**); 0.5 mm (**D**); 20 µm (**E–I**); 10 µm (**J, K**).

##### Cultures and anamorph.

**Figure 6.**
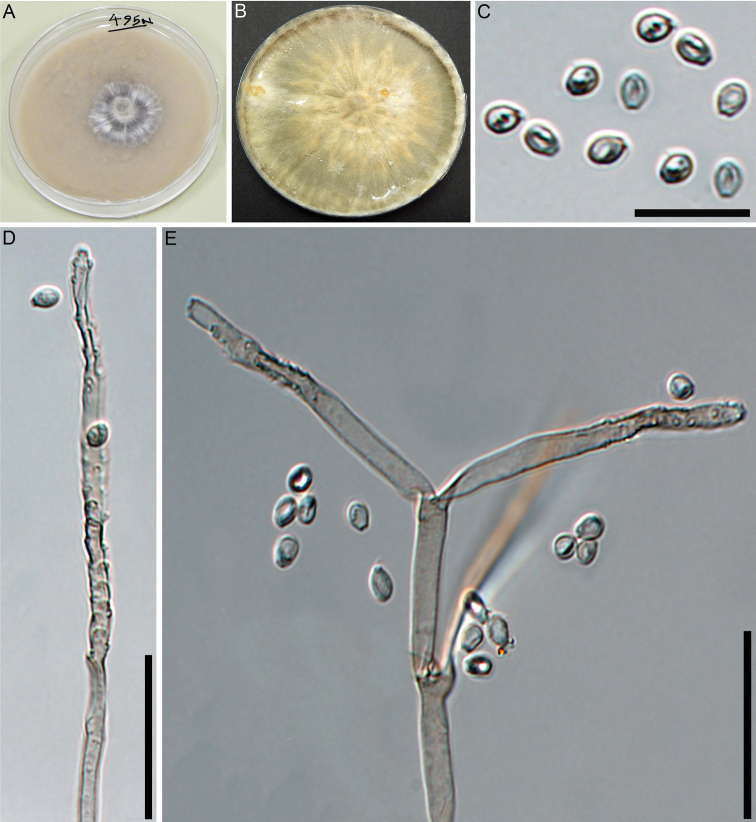
Culture and anamorphic structures of *Nemaniaserpens* (MUCL 57702) on OA **A, B** surface of colony after (**A**) 7 and (**B**) 50 days of incubation of *N.serpens***C** conidia of *N.serpens***D, E** general view of anamorph structure, conidiophores, conidiogenous cells and mature conidia of *N.serpens*. Scale bars: 10 µm (**C**); 20 µm (**D, E**).

Colonies on OA covering a 9 cm Petri dish in 18 days, at first white becoming Vinaceous (57), felty, azonate; finally, attaining Amber (47) to Honey (64) after 50 days. Anamorph geniculosporium-like. Conidiophores variables in length, hyaline to light brown. Conidiogenous cells up to 60 × 2.5–3.2 µm, hyaline to light brown. Conidia hyaline, ellipsoid with truncate base, 3–4.8 × 2–3.5 µm (Fig. 6).

##### Specimens examined.

Iran, Mazandaran Province, Ramsar County, Safarud Forest, 36°53'49"N, 50°35'29"E, 815 m elev., on fallen branch of *Parrotiapersica*, 29 October 2016, leg. M.J. Pourmoghaddam (GUM 1625; living culture MUCL 57702, IRAN 3735C); Guilan Province, Astara County, 38°23'04"N, 48°51'45.10"E, 1 m elev., on fallen branch of *Parrotiapersica*, 22 October 2021, leg. M.J. Pourmoghaddam (GUM 1903).

##### Notes.

**Figure 7.**
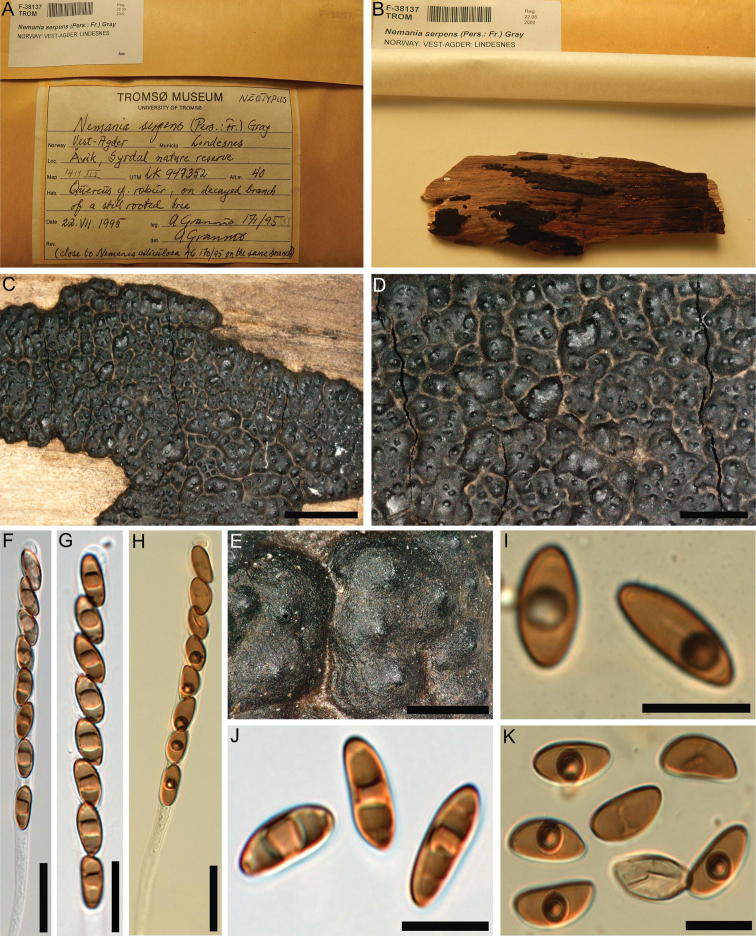
*Nemaniaserpens* (neotype) **A** herbarium label **B** stromata on wood **C, D** close-up view of stroma surface **E** close-up view of stroma surface showing ostioles **F, G** mature ascus in water **H** mature ascus in Melzer’s reagent, showing the inamyloid (not bluing) ascal apical apparatus **I** ascospores in water showing straight germ slit much less than spore-length **J, K** ascospores in water. Scale bars: 3 mm (**C**); 1 mm (**D**); 0.5 mm (**E**); 20 µm (**F–H**); 10 µm (**I–K**).

*Nemaniaserpens* is a very common fungus in Europe (Petrini and Rogers 1986; http://pyrenomycetes.free.fr/, accessed 8 Aug 2022). In combination with pale olive brown ascospores with broadly rounded ends and with a short inconspicuous germ slit, *N.serpens* is characterised by a dextrinoid reaction of the ascal apical apparatus in Lugol’s solution, while it does not react in Melzer’s reagent, which is an exceptional combination within *Nemania* (Granmo et al. 1999; http://pyrenomycetes.free.fr/, accessed 8 Aug 2022). Most of the characters of the Iranian specimens are in accordance with the neotype specimen (Fig. 7; Ju and Rogers 2002), aside from insignificant variations in the size of ascospores. We studied the neotype material and did not observe a conspicuous ascal apical apparatus as described by Ju and Rogers (2002). Morphological species identification of the Iranian specimens is corroborated by the ITS sequence data, as the Iranian and the ex-neotype sequence of *N.serpens* are almost identical (3 substitutions, 3 gaps). Finally, we would like to mention that, for the neotype specimen, Daranagama et al. (2018) erroneously described the ascal apical apparatus as bluing (I+) in Melzer’s reagent, while their fig. 7h clearly shows a not bluing (I-) ascal apical apparatus.

#### ﻿ Nemania diffusa

Taxon classificationFungiXylarialesXylariaceae

(Sowerby) S.F. Gray, Nat. Arr. Brit. Pl. (London) 1: 517 (1821).

C3A1BE94-0A03-5B33-9EBD-A0708C4B08A4

Fig. 8

##### Teleomorph.

Stromata superficial, effused-pulvinate, discoid, up to 2 cm long × 0.3–1.5 cm wide, sessile, attachment to substrate with narrow connective; surface dark brown to blackish-brown, with inconspicuous perithecial mounds, carbonaceous immediately beneath surface; tissue between and beneath perithecia black to dark brown. Perithecia obovoid to cylindrical, 0.5–0.8 mm high × 0.3–0.5 mm wide. Ostioles papillate to coarsely papillate. Asci cylindrical, with amyloid, urn-shaped apical apparatus, 2–3 µm high × 1.5–2 µm wide, stipe up to 100 µm long, spore-bearing part 70–80 × 7–10 µm. Ascospores smooth, unicellular, brown to dark brown, ellipsoid, inequilateral, with narrowly rounded ends, 9.5–13(–14) × 4.5–6.5 µm, with straight germ slit spore-length on flattened side; perispore indehiscent in 10% KOH.

##### Specimen examined.

Iran, Guilan Province, Rezvanshahr County, 37°37'52"N, 40°02'18"E, 7 m elev., on fallen branch of *Quercuscastaneifolia*, 6 October 2016, leg. M.J. Pourmoghaddam (GUM 1626), ITS and LSU sequences GenBank OP352258 and OP352270, respectively.

##### Notes.

*Nemaniadiffusa*, originally described from England (Sowerby 1803), is a widespread and fairly common species in Europe (Fournier et al. 2018). It has also been reported from North and South America (Petrini and Rogers 1986), Papua-New Guinea (Van der Gucht 1995) and Taiwan (Ju and Rogers 1999), but it has yet to be proven whether all these morphologically identified accessions are conspecific with the European ones. The Iranian specimen is in accordance with previous descriptions by Ju and Rogers (2002). It can be differentiated from *N.albocincta* by its larger ascospores [9.5–13.5 × (4.5–)5–6 vs. 8–10 × 4–5 µm], which are also more equilateral. *Nemaniaobscura* also differs from it in stromatal features and smaller, strongly inequilateral ascospores (8.2–9.4 × 4.5–5.3 µm) with subacute ends. Despite several attempts, we could not achieve a living culture. Therefore, to confirm our morphological species identification, we extracted DNA from stromata and performed PCR (ITS/LSU) and sequencing according to Pourmoghaddam et al. (2018). The ITS sequence of the Iranian collection (OP352258) is completely identical to numerous sequences of European accessions of *N.diffusa*, some of which are morphologically well-documented to represent the species (e.g. MW489542 from Switzerland; Senn-Irlet et al. 2021), confirming the species identification. However, as *RPB2* and *TUB2* could not be obtained, the Iranian accession of *N.diffusa* was not added to the phylogenetic multi-locus analyses.

**Figure 8.**
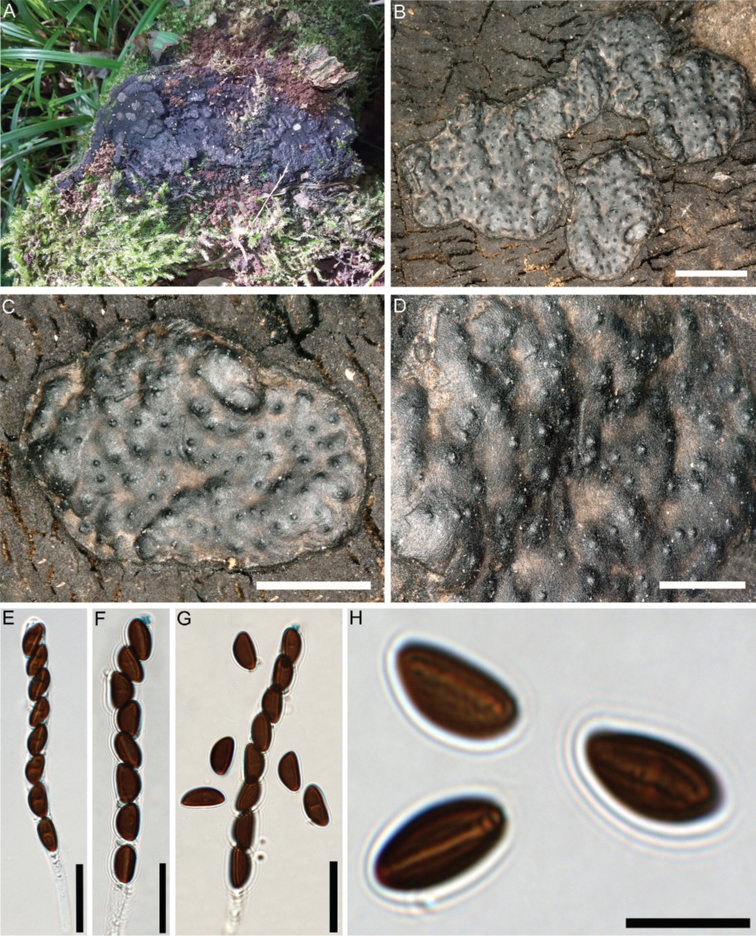
*Nemaniadiffusa* (GUM 1626) **A** stromatal habit **B** close-up view of stromatal surface **C, D** close-up view of stroma surface showing ostioles **E** mature ascus in water **F, G** mature asci in Melzer’s reagent showing the amyloid (bluing) ascal apical apparatus **H** ascospore showing straight germ slit. Scale bars: 3 mm (**B**); 1.5 mm (**C**); 0.8 mm (**D**); 20 µm (**E–G**); 10 µm (**H**).

## ﻿Discussion

In this study, we examined the phylogenetic relationships of our fresh collections with all species of *Nemania* for which multigene sequence data are available. We have performed a multigene analysis using ITS, LSU, *RPB2* and *TUB2* sequence data to determine the phylogenetic placement of these species. *Nemania* (including *Euepixylon*) clearly forms a monophyletic clade in the phylogenetic analysis which has been placed in Xylariaceae for a long time (Hyde et al. 2020). The results of our phylogenetic analyses agree well with those of Pi et al. (2021), their clade N6 corresponding to our clade N1, their clade N5 to our clade N2 and their clades N1–4 to our clade N3.

Remarkably, in the phylogenetic analyses, the two previously-accepted species of *Euepixylon* are not only contained within *Nemania*, but also do not form a monophyletic lineage, yet they are members of the same *Nemania* subclade 1 (N1; Fig. 1). Stroma morphology and the anamorph of *Euepixylon* matches *Nemania*, the main distinguishing feature being poroid (*Euepixylon*) vs. straight, conspicuous or inconspicuous germ slits of variable length (*Nemania*; Læssøe and Spooner 1993, Granmo et al. 1999). When re-establishing the genus *Euepixylon*, already Læssøe and Spooner (1993) doubted whether the genus will survive in the long run. Considering the results of the phylogenetic analyses, germ site morphology is clearly not a good character to separate *Euepixylon* from *Nemania* and the former genus should be considered as a synonym of the latter, which has already been implemented by for example, Pi et al. (2021) and Voglmayr et al. (2022) and which we, therefore, also adopt here. Synonymy of both genera is further supported by the fact that the type species of *Euepixylon* (*E.udum*), as well as *Nemania* (*N.serpens*), are revealed to be closely related within the *Nemania* subclade 1 (N1).

Most *Nemania* species are morphologically highly similar, which makes species delimitation and identification based on morphology alone difficult and confusing (Granmo et al. 1999; Ju and Rogers 2002; Fournier et al. 2018). Recently, much progress in reliable species identification has been achieved by DNA sequence data, particularly protein-coding genes such as *RPB2* or *TUB2*, which have superior resolution compared to ITS or LSU (Lücking et al. 2020; Stadler et al. 2020). However, an obstacle for an improved species delimitation and classification is the lack of sequences of type material or well-identified reference specimens in GenBank, which is particularly important for morphologically difficult and complex lineages. *Nemaniaserpens*, the type species of the genus, is a good example of these problems. Until the present study, no verified sequence data were available in GenBank for *N.serpens* and the various accessions deposited under this name do not form a monophylum in phylogenetic analyses (data not shown). However, it has been widely ignored that Granmo et al. (1999), who neotypified *N.serpens* with a recent Norwegian collection (TROM 174), also generated and published an ITS sequence of their neotype. The reason for disregarding this ex-neotype ITS sequence in subsequent studies lies the fact that Granmo et al. (1999) published their sequences in their Appendix 3, a colour figure of the ITS alignment they used for their phylogenetic analyses, but they did not deposit them in a public sequence repository. The ITS sequences of Granmo et al. (1999) can, therefore, only be added to a sequence matrix if they are transcribed from this colour figure alignment, which we have done here. The addition of the ex-neotype ITS sequence of *N.serpens* from Granmo et al. (1999) to our sequence matrix revealed a high similarity to our Iranian isolate that was identified as *N.serpens* by morphological comparison with the neotype specimen. The phylogenetic analyses also revealed that another isolate (HAST 235), commonly included as *N.serpens* in phylogenies, is not closely related to the neotype, but forms a highly-supported clade with another species, *N.chestersii*, which indicates that HAST 235 does not represent *N.serpens*, but is misidentified.

A further example for incorrectly labelled sequences that could be clarified by inclusion of the ITS sequences of Granmo et al. (1999) refers to CBS 679.86, another accession erroneously deposited as *N.serpens* in GenBank. In the phylogenetic analyses, the accession CBS 679.86 has an ITS sequence almost identical to that of the ex-holotype sequence of *N.prava* from Granmo et al. (1999). However, this becomes conclusive considering that culture CBS 679.86 represents an ex-paratype culture of Hypoxylonatropurpureumvar.brevistipitatum (Petrini and Rogers 1986), a synonym of *Nemaniaprava* (Granmo et al. 1999). Granmo et al. (1999) confirmed this synonymy by revealing identical ITS sequences for the holotype of *N.prava*, the holotype of Hypoxylonatropurpureumvar.brevistipitatum and another paratype of the latter. It remains yet unclear why the sequences of culture CBS 679.86 have been deposited as *N.serpens* in GenBank. These exemplary cases once again demonstrate that species names of GenBank sequences, as well as the sources of the sequence data, need to be critically evaluated, in particular in taxonomically difficult groups.

Stromata of *Nemania* are highly carbonised and do not contain large amounts of secondary metabolites, as is the case in other phylogenetically closely-related genera, such as *Dematophora* and *Rosellinia*. Only small amounts of xylaral (in *N.diffusa*; Stadler et al. 2008) and BNT (in young *Nemania* specimens; Stadler and Hellwig 2005) have so far been detected.

Since the cultures of Xylariaceae are, in general, rich in production of secondary metabolites (Helaly et al. 2018; Becker and Stadler 2021), further analysis of *Nemania* species may be useful for a better taxonomic classification in the future. Chestersiene and furanone production have so far been described as characteristic metabolites, delimiting *Nemania* from *Hypoxylon* (Whalley and Edwards 1995). Even though this work was based on strains that are apparently not deposited in public collections, the respective compounds have, indeed, not been found in any other fungal genus. The lack of extant cultures for many described xylariaceous species, including for example, *Rosellinia* and *Dematophora* (cf. Wittstein et al. 2020), precludes comprehensive chemotaxonomic studies in the family. Recent progress in the generation of high-quality genome sequences could also enable the search for possible discriminatory biosynthetic gene clusters, as presence or absence of a cluster can serve as a predictor of the taxonomic relationship, which might be an option for future comprehensive sequencing campaigns (Wibberg et al. 2021; Kuhnert et al. 2021).

Xylariaceae is one of the most important ascomycete families found in the north of Iran which has regions with subtropical climates and houses numerous species. Until recently, studies on species biodiversity of Xylariaceae focused on the genera *Xylaria* (Hashemi et al. 2014, 2015), *Kretzschmaria* (Pourmoghaddam et al. 2018) and *Rosellinia* (Pourmoghaddam et al. 2022), which we here extend to the genus *Nemania*.

## Supplementary Material

XML Treatment for Nemania hyrcana

XML Treatment for Nemania serpens

XML Treatment for Nemania diffusa

## Supplementary materials

Supplementary material 1 Alignment Data type Nex file. https://binary.pensoft.net/file/753838This dataset is made available under the Open Database License (http://opendatacommons.org/licenses/odbl/1.0/). The Open Database License (ODbL) is a license agreement intended to allow users to freely share, modify, and use this Dataset while maintaining this same freedom for others, provided that the original source and author(s) are credited.Mohammad Javad Pourmoghaddam, Christopher Lambert, Hermann Voglmayr, Seyed Akbar Khodaparast, Irmgard Krisai-Greilhuber, Marc Stadler
